# Fast and Slow Oscillations Recruit Molecularly-Distinct Subnetworks of Lateral Hypothalamic Neurons *In Situ*

**DOI:** 10.1523/ENEURO.0012-18.2018

**Published:** 2018-02-07

**Authors:** Christin Kosse, Denis Burdakov

**Affiliations:** Neurophysiology Laboratory, The Francis Crick Institute, London NW1 1AT, United Kingdom

**Keywords:** electrophysiology, hypocretin, hypothalamus, MCH, orexin, oscillations

## Abstract

Electrical signals generated by molecularly-distinct classes of lateral hypothalamus (LH) neurons have distinct physiological consequences. For example, LH orexin neurons promote net body energy expenditure, while LH non-orexin neurons [VGAT, melanin-concentrating hormone (MCH)] drive net energy conservation. Appropriate switching between such physiologically-opposing LH outputs is traditionally thought to require cell-type-specific chemical modulation of LH firing. However, it was recently found that, *in vivo,* the LH neurons are also physiologically exposed to electrical oscillations of different frequency bands. The role of the different physiological oscillation frequencies in firing of orexin vs non-orexin LH neurons remains unknown. Here, we used brain-slice whole-cell patch-clamp technology to target precisely-defined oscillation waveforms to individual molecularly-defined classes LH cells (orexin, VGAT, MCH, GAD65), while measuring the action potential output of the cells. By modulating the frequency of sinusoidal oscillatory input, we found that high-frequency oscillations (γ, ≈30–200 Hz) preferentially silenced the action potential output orexin_LH_ cells. In contrast, low frequencies (δ-θ, ≈0.5–7 Hz) similarly permitted outputs from different LH cell types. This differential control of orexin and non-orexin cells by oscillation frequency was mediated by cell-specific, impedance-unrelated resonance mechanisms. These results substantiate electrical oscillations as a novel input modality for cell-type-specific control of LH firing, which offers an unforeseen way to control specific cell ensembles within this highly heterogeneous neuronal cluster.

## Significance Statement

Neurons that emit opposing control signals are physically intermixed in the mammalian hypothalamus. How to achieve selective control of particular hypothalamic signals remains a fundamental unsolved question in both basic science and clinical practice. Traditionally, such cell-selective control is only thought to be possible by chemicals, while electricity is assumed to be too nonselective. We investigated this assumption by exploring the effects of biologically-relevant electrical oscillations on signals emitted by specific types of hypothalamic cells. Unexpectedly, some oscillation frequencies switched off certain hypothalamic signals (orexin signals) while preserving others (non-orexin signals), via a cell-selective resonance mechanism. These findings increase our understanding of how the hypothalamus can be controlled.

## Introduction

The lateral hypothalamus (LH) controls states of consciousness, energy balance, and motivated behavior in mammals (Saper et al., 2001; Stuber and Wise, 2016). Several molecularly and physiologically -distinct types of LH neurons exist. Orexin/hypocretin_LH_ neurons promote generalized (cortical and sympathetic) arousal and net energy use; their hypoactivity evokes energy conservation, weight gain, and pathologic intermixing of sleep and wakefulness, and their hyperactivity is associated with stress and anxiety (Chemelli et al., 1999; Hara et al., 2001; Boutrel et al., 2005; Suzuki et al., 2005; Adamantidis et al., 2007; Sakurai, 2014; Bonnavion et al., 2015). VGAT_LH_ neurons promote consumptive behaviors leading to net energy gain: their hypoactivity reduces body weight, eating, and arousal; and their hyperactivity promotes overeating and hyperarousal (Jennings et al., 2015; Herrera et al., 2016; Venner et al., 2016). Melanin-concentrating hormone (MCH_LH_) neurons promote memory-associated brain states, suppress locomotion, and promote weight gain (Shimada et al., 1998; Jego et al., 2013; Whiddon and Palmiter, 2013). GAD65_LH_ neurons, which do not overlap with the orexin_LH_ and MCH_LH_ neurons and only partly overlap with VGAT_LH_ cells, are necessary and sufficient for normal locomotion (Kosse et al., 2017).

Differential control of these physiologically-distinct LH drives is presumably required for proper physiological regulation and for avoiding co-occurrence of contradictory drives. This control has been a subject of intense research, revealing cell-type-specific modulation of the LH by chemical agents such as nutrients, hormones, neurotransmitters, and gasses (Li et al., 2002; Yamanaka et al., 2003; van den Pol et al., 2004; Burdakov et al., 2005; Williams et al., 2007; Karnani et al., 2011; Burdakov et al., 2013; Karnani et al., 2013). Apart from these chemical signals, many brain regions, including the LH, contain electrical oscillations (Gray and Singer, 1989; Salinas and Sejnowski, 2001; Buzsáki and Draguhn, 2004; Carus-Cadavieco et al., 2017). These oscillations are thought to control brain states and behaviors in a frequency-dependent manner, for example, fast oscillations (γ frequencies, ≈30–200 Hz) orchestrate arousal, memory, sensory processing, and decision-making (Cardin et al., 2009; Colgin et al., 2009; Buzsáki and Wang, 2012; Yamamoto et al., 2014). In the LH, γ oscillations, controlled in part by inputs from the lateral septum, were recently found to be associated with food approach behavior, and differentially affect subthreshold membrane potential of MCH_LH_ and VGAT_LH_ cells (Carus-Cadavieco et al., 2017). However, it remains unknown whether different oscillations frequencies differentially modulate the physiologic output (action potential firing rate) of orexin versus non-orexin neurons.

Oscillations shape the synaptic inputs onto individual neurons, which collectively results in sinusoidal oscillations of current input at varying frequencies in neurons recorded intracellularly *in vivo* (Leung and Yim, 1986; Soltesz and Deschênes, 1993). Neurons control long-range targets by action potentials fired in response to the input signals. Understanding how the firing rates of molecularly-defined LH neurons respond to oscillatory input currents may thus reveal a new dimension of LH output tuning and input-output information transfer. Using experimental paradigms established for studying the effects of oscillations on neuronal firing in other brain regions (Pike et al., 2000), here we explored how the firing of individual, molecularly-defined LH neurons is modulated by the frequency of oscillatory current inputs.

## Materials and Methods

### Identification of molecularly-distinct cell classes by transgenic labeling

All procedures followed United Kingdom Home Office regulations and were approved by local welfare committees. Adult male and female mice (at least eight weeks old) were kept on a standard 12/12 h light/dark cycle and on standard mouse chow and water *ad libitum*. Mice were anaesthetized with isoflurane and injected with Meloxicam (2 mg/kg bodyweight, s.c.) for analgesia. After placing into a stereotaxic frame (David Kopf Instruments), a craniotomy was performed and a borosilicate glass pipette was used to inject viral vectors into the LH bilaterally with pressure (coordinates AP/DV/ML = −1.3/−5.15 to −5.25/1.0, −1.0 mm; infusion speed = 75 nl/min, injection volume 75 nl). Mice were allowed to recover for at least two weeks after surgery while single housed. To study LH MCH cells, we labeled them with mCherry by injecting into the LH a lentiviral vector specifically expressing mCherry in MCH neurons VSVG.HIV.MCH.mCherry(p2428) (3.16 × 10^−11^ gc/ml, vector described and validated in Apergis-Schoute et al., 2015). To study LH orexin neurons, we used either the previously characterised and validated orexin-eGFP mice (Burdakov et al., 2006), or labeled LH orexin cells by injecting into the LH an orexin promoter-dependent adeno-associated vector specifically expressing GCaMP6s in orexin cells (AAV1.hORX.GCaMP6s.hGH, 2.53 × 10^−12^ gc/ml, vector described and validated in González et al., 2016). To study LH VGAT or GAD65 cells, we used GAD65-GFP mice (Karnani et al., 2013) crossed with VGAT-Ires-Cre mice (Vong et al., 2011) and CAG-tdTomato mice (Madisen et al., 2010); and made recordings from GFP(+)/tdTomato(-) or GFP(-)/tdTomato(+) cells that corresponded to GAD65(+)/VGAT(-) and VGAT(+)/GAD65(-) cells, respectively (Kosse et al., 2017).

### Chemicals and solutions

For brain slice recordings, artificial CSF (ACSF) and ice-cold slicing solution were gassed with 95% O_2_ and 5% CO_2_, and contained the following ACSF: 125 mM NaCl, 2.5 mM KCl, 1 mM MgCl_2_, 2 mM CaCl_2_, 1.2 mM NaH_2_PO_4_, 21 mM NaHCO_3_, 2 mM D-(+)-glucose, 0.1 mM Na+-pyruvate, and 0.4 mM ascorbic acid. Slicing solution: 2.5 mM KCl, 1.3 mM NaH_2_PO.H_2_O, 26.0 mM NaHCO_3_, 213.3 mM sucrose, 10.0 mM D-(+)-glucose, 2.0 mM MgCl_2_, and 2.0 mM CaCl_2_. For standard whole-cell recordings, pipettes were filled with intracellular solution containing the following: 120 mM K-gluconate, 10 mM KCl, 10 mM HEPES, 0.1 mM EGTA, 4 mM K_2_ATP, 2 mM Na_2_ATP, 0.3 mM Na_2_GTP, and 2 mM MgCl_2_, pH 7.3 with KOH. All chemicals were from Sigma or Tocris Bioscience.

### Acquisition and analysis of electrophysiological data

Standard whole-cell slice patch-clamp recordings were conducted as described in detail in our previous studies (Schöne et al., 2014). Briefly, LH slices were prepared at least two weeks after the virus injection. After gluing a block of brain with cyanoacrylate glue to the stage of a Campden Vibroslice, coronal brain slices (250-µm thickness) containing the LH were cut while immersed in ice-cold slicing solution. Slices were incubated for 1 h in ACSF at 35°C, then transferred to a submerged-type recording chamber. Living neurons containing fluorescent markers were visualized in acute brain slices with an upright Olympus BX61WI microscope equipped with an oblique condenser and appropriate fluorescence filters. Data were acquired with HEKA Patchmaster and analyzed with HEKA Fitmaster, GraphPad Prism and Matlab.

To determine the frequency preference for action potential firing, a protocol of 5-s-long sinusoidal currents at the following fixed frequencies was applied: 0.5, 1, 2, 3, 5, 7, 10, 15, 20, 30, 50, 70, 100, and 200 Hz. A minimum of 20-s stable baseline recording was obtained before the sinusoidal stimulations, and the stimulations were applied with an interval of 5 s. Membrane time constants (τ) were calculated from fitting a single exponential function to the initial part of a voltage response to a small hyperpolarising current pulse, and input resistances (*R_i_*) were derived from Ohm’s law by fitting a linear function to the current−voltage relationship of voltage responses to hyperpolarising current pulses (Table 1). From these values, the input frequency (*f*) dependence of membrane impedance (*Z*) was calculated as follows (based on Gutfreund et al., 1995; Pike et al., 2000):

|Z|=Ri(τ2(2πf)2+1)

**Table 1.** Passive electrical properties of neurons analyzed in this study

	MCH	Orexin	GAD65	VGAT
Membrane resistance (MΩ)	474.8571 ± 54.2614, *n* = 14	463.0385 ± 56.76324, *n* = 13	619.2471 ± 49.09955, *n* = 17	724.4438 ± 92.54686, *n* = 16
Membrane time constant (ms)	40.60857 ± 6.530006, *n* = 14	43.24915 ± 7.335909, *n* = 13	32.42376 ± 4.217719, *n* = 17	32.54188 ± 3.505608, *n* = 16

### Experimental design and statistical analysis

Cells were randomly recorded throughout the anatomic extent of the LH, by choosing fluorescent neurons using an objective that blinded the experimenter to intra-LH location of the cell due to its small field of view (a high-magnification 40× objective). After recording, the intra-LH locations of recorded neurons were confirmed using a large-field (low magnification) objective. Statistical tests and descriptive statistics were performed as stated in the figure legends. Before performing parametric tests, data were assessed for normality with a D’Agostino–Pearson omnibus test or Kolmogorov–Smirnov test and variances were assessed for homogeneity with a Brown–Forsythe test. To compare interactions within data with repeated measurements, ANOVA was used, and if significant interactions were found, multiple comparison tests followed. Normalizations were performed on a single cell basis by dividing by the largest value obtained per cell. Cells were deemed active if a paired *t* test comparing normalized firing and impedance values was significant after controlling for the false discovery rate (which was set to 5%) by a two-stage step-up method of Benjamini, Krieger, and Yekutieli. Analysis was performed with GraphPad Prism and Matlab.

## Results

### Distinct frequency preferences of molecularly-distinct LH subnetworks

**Figure 1.**
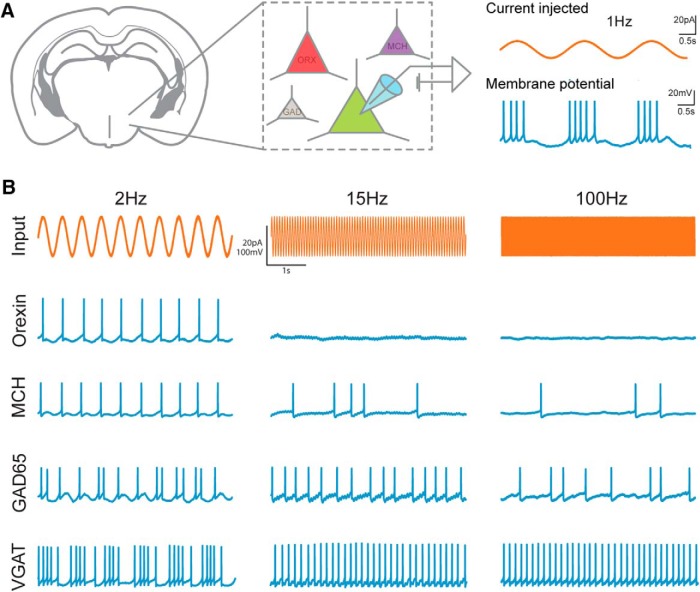
***A***, Overview of experimental strategy. Cell types were genetically tagged with a fluorophore to target patch-clamp recordings. During whole-cell recordings, 5-s-long oscillatory current at fixed frequencies were injected into the cells to obtain an action potential output corresponding to each frequency. ***B***, Individual example raw traces of single cells of the investigated cell types at three different input frequencies.

To explore how different LH neurons respond to oscillatory inputs, we selectively targeted fluorescent reporters to LH orexin, VGAT, MCH, or GAD65 cells (see Materials and Methods) and recorded the membrane potential responses of individual genetically-defined LH cells to sinusoidal input currents at a broad range of physiological frequencies (0.5–200 Hz; Fig. 1). To facilitate comparisons between neurons, and to previous studies of neuronal responses to oscillations in other brain areas (Pike et al., 2000), the recordings were performed at the membrane potentials close to threshold for spike generation. This was achieved by superposing an oscillatory current on the maximum step current that itself did not elicit spikes, and using a small (20 pA) peak-to-peak sinusoidal current (based on Pike et al., 2000).

**Figure 2.**
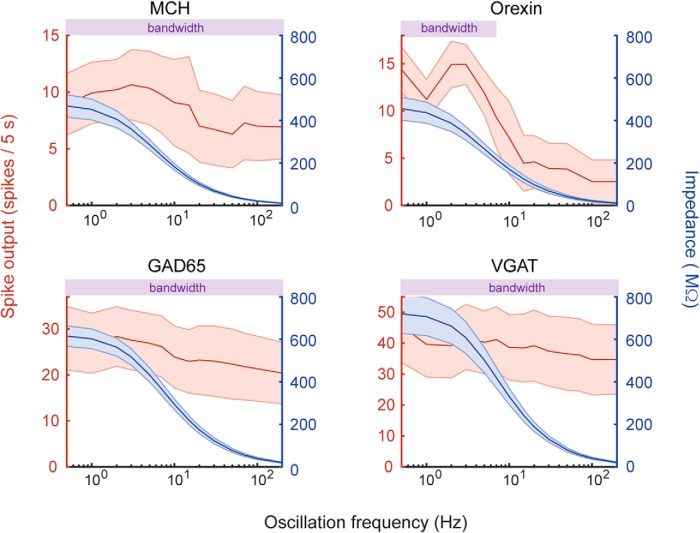
Effects of input oscillation frequency on LH cell population firing rates (spike output, red) and impedances (blue). Bandwidth bars (purple) denote oscillation frequencies at which there was significant (non-zero) spike output (calculated by one-sample *t* tests and corrected for multiple comparisons by controlling the false discovery rate, see Materials and Methods). Values are mean ± SEM. Cell numbers for MCH, orexin, GAD65, and VGAT neurons are 14, 13, 17, and 16, respectively.

Low input frequencies (≈0.5–20 Hz) resulted in robust spiking activity in all LH neuronal types (Figs. 1, 2). In contrast, higher frequencies selectively silenced orexin neurons (cessation of significant firing at inputs above 7 Hz; Fig. 2*A*), while preserving significant firing in non-orexin cell types (Fig. 2*B–D*). These differences in frequency-preferences of LH neuron firing did not appear to be related to their maximal firing rates or spike-rate adaptation. Specifically, the firing of non-orexin neurons stayed relatively invariant across oscillation frequencies, irrespective of whether their maximal firing rates were fast (VGAT, GAD65 cells) or slow (MCH cells), and irrespective of whether their spike-rate adaptation was high (MCH cells; van den Pol et al., 2004; Burdakov et al., 2005) or low (GAD65 cells; Karnani et al., 2013). In turn, orexin cell firing had higher frequency-dependent decay than non-orexin cell firing, although their initial firing was faster than MCH cells but slower than VGAT or GAD65 cells (Fig. 2), and their spike-rate adaptation was lower than that of MCH cells (Burdakov et al., 2005). Thus, there are distinct frequency-bandwidths for optimal firing of orexin and non-orexin LH neurons, which cannot be accounted for by previously-studied differences in their intrinsic excitability.

These distinct frequency dependencies of firing in orexin and non-orexin neurons could, in theory, emerge from distinct frequency dependencies of the passive membrane impedances (Pike et al., 2000). Higher membrane impedance would produce greater membrane potential fluctuations in response to oscillatory inputs and thus produce greater membrane excitation and firing (Pike et al., 2000). To investigate whether such passive membrane resonance could account for the differences in spike frequency preferences (Fig. 2, red plots), we used our data to compute impedances of RC equivalent circuits at each input frequency for individual LH neurons (Fig. 2, blue plots; see Materials and Methods). Although maximum impedances differed between cell types (orexin = MCH < GAD65 < VGAT neurons; Fig. 2), all impedances decayed similarly with input oscillation frequency, and this decay and did not follow the associated frequency-tuning of firing (Fig. 2, compare red and blue plots).

**Figure 3.**
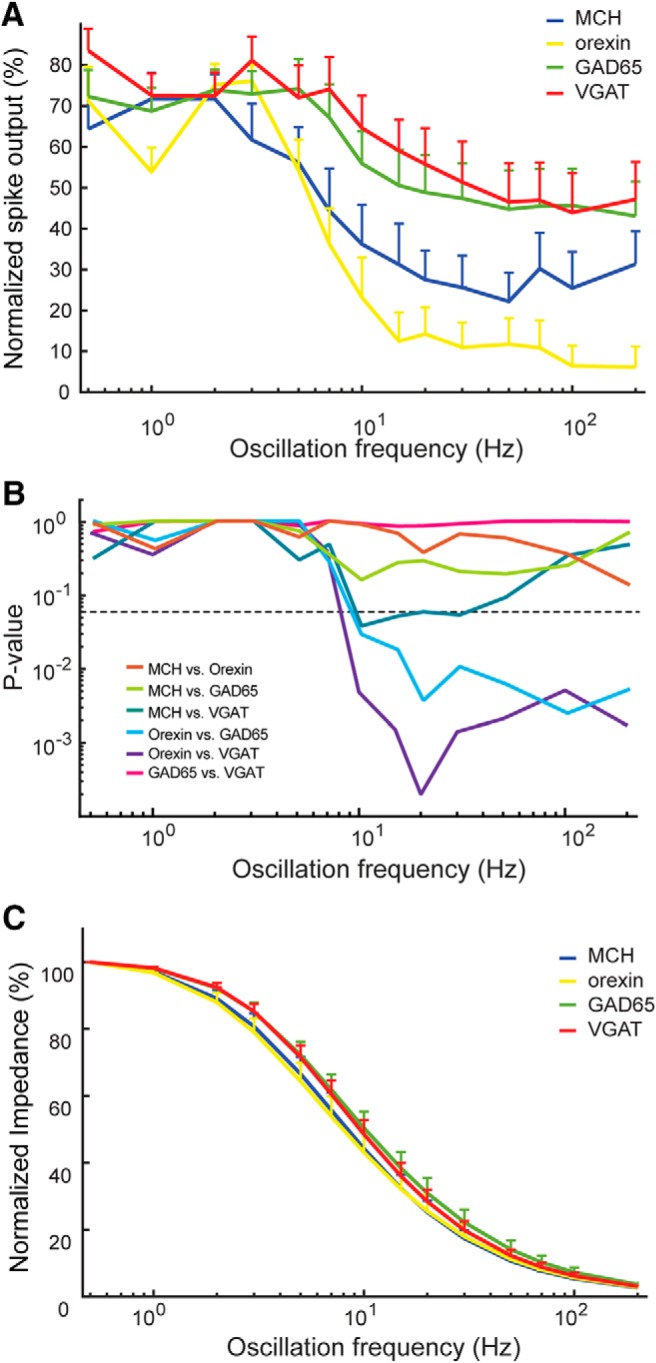
***A***, Effect of input oscillation frequency on LH cell population spike outputs (same spike data as in Fig. 2, but normalized to the maximum spike output in each cell, to facilitate comparisons of oscillation frequency effects between cell classes). Values are mean ± SEM. ***B***, Differences in population spike outputs across oscillation frequencies. The *y*-axis shows adjusted *p* values of a two-way repeated-measures ANOVA with Tuckey’s multiple comparison correction, for the four cell types and 14 input oscillation frequencies (ANOVA: interaction, *F*_(36,672)_ = 1.907; *p* = 0.0013). ***C***, Effect of input oscillation frequency on LH cell population membrane impedances (same impedance data as in Fig. 2, but normalized to the maximum impedance in each cell). Values are mean ± SEM.

To compare the frequency-tuning of firing and impedances between different LH cell types, independently of differences of absolute values in these parameters, we normalized each neuron to its own maximal firing and impedance (Fig. 3; see Materials and Methods). Similar to raw data (Fig. 2), this revealed that orexin cell firing decayed more steeply with oscillation frequency than that of non-orexin cells (Fig. 3*A*; within these normalized data, the decay was significantly different between orexin and VGAT or GAD65 cells, but not between orexin and MCH cells; Fig. 3*B*). This difference between orexin and VGAT/GAD65 cells emerged sharply at >7 Hz and persisted at higher frequencies (Fig. 3*B*). In contrast, there was an almost perfect overlap in the frequency dependence of normalized membrane impedances in the four LH cell types (Fig. 3*C*).

### Frequency preference variation of individual cells within each molecularly-defined subnetwork

**Figure 4.**
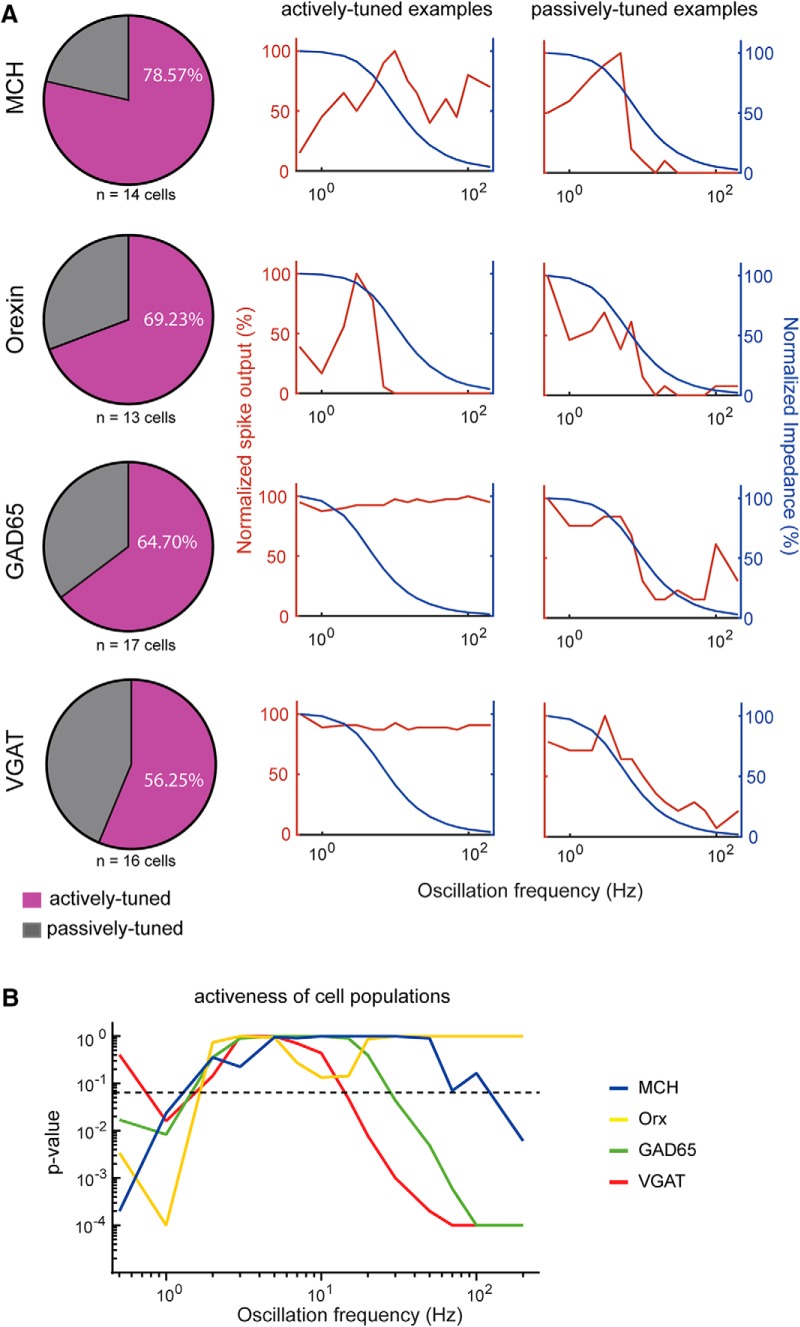
***A***, left, Pie charts depicting the percentage of actively-tuned cells (cells whose normalized spike frequency significantly differs from its normalized impedance magnitude), and passively-tuned cells (cells whose normalized spike frequency did not significantly differ from its normalized impedance magnitude). To group neurons into these categories, firing and impedance profiles of each individual cell were compared using a paired *t* test with correction for multiple comparisons by controlling the false discovery rate (two-stage step-up method of Benjamini, Krieger, and Yekutieli). Middle, Examples of individual actively-tuned cells. Right, Examples of individual passively-tuned cells. ***B***, Activeness of cell populations (statistical difference between normalized spike frequency and normalized impedance of each cell type, across input oscillation frequencies, n numbers for each cell type are as indicated in ***A***. The *y*-axis shows adjusted p-values from paired *t* tests with correction for multiple comparisons by controlling the false discovery rate (two-stage step-up method of Benjamini, Krieger, and Yekutieli).

We next investigated the differences between the impedance-predicted and experimentally-observed frequency-tuning of LH cell firing at the level of individual neurons. Within each molecularly-distinct class, individual neurons displayed similar frequency-tuning of impedance (as was the case also between classes; Fig. 3*C*), but differed substantially in frequency-tuning of firing (Fig. 4*A*). As the input oscillation frequency increased, the firing rate decay mirrored the impedance decay in some cells (Fig. 4*A*, typical examples in right column), but strikingly deviated from impedance in other cells (Fig. 4*A*, typical examples in middle column). By quantifying and analyzing the difference between normalized impedance and firing in each cell (see Materials and Methods), we estimated, within each cell type, the percentage of cells that were tuned passively (i.e. firing tuning similar to impedance tuning) or actively (firing tuning significantly deviating from impedance tuning; Fig. 4*A*, left column). This revealed that within each cell type, the majority of cells were actively tuned, but some cell classes contained more “active” cells than others (MCH > orexin > GAD65 > VGAT; Fig. 4*A*).

Finally, we analyzed how “cell activeness” (difference between observed and impedance-predicted firing) varies as a function of input oscillation frequency within each cell type (Fig. 4*B*). Active tuning (significant difference between observed and impedance-predicted firing) was present in all cell types at low frequencies (<1 Hz), where firing was lower than expected from impedance (Fig. 4*B*). However, as input oscillation frequency increased, the frequency dependence of orexin population firing became indistinguishable from the frequency dependence of orexin cell impedance, with both sharply decaying as oscillation frequency increased (Fig. 4*B*). In contrast, VGAT and GAD65 populations (and to a lesser extent the MCH population) maintained substantial firing in the γ-fast frequency range (30–200 Hz; Fig. 4*B*). Thus, orexin neuron firing is subject to steep impedance-associated decay during γ input, but non-orexin neurons resist this decay and maintain firing during γ input.

## Discussion

Cell-type-specific control of LH firing is important for normal physiology (avoiding contradictory LH outputs), for basic research (studying the role of specific LH outputs), and potentially for clinical applications (controlling sleep and appetite in obese or insomniac patients). In this study, we found that such cell-type-specific control can, unexpectedly, be achieved by varying the frequency of electrical oscillations in the LH. Specifically, we found significant differences in frequency dependence of orexin and non-orexin cell firing (Fig. 2), which were especially striking in orexin versus VGAT or GAD65 neurons (Fig. 3*B*), and were not explained by cell-type-specific variation in passive membrane impedances (Fig. 3*C*). Thus, distinct cell classes in the LH network show distinct frequency preferences for spike generation. Orexin neurons show a preference for low frequencies (<10 Hz), while non-orexin neurons are significantly driven by low and high (10–200 Hz) frequencies.

The monotonic decay in membrane impedance that occurs as oscillation frequency is increased would be expected to produce concurrent monotonic decay in firing (Pike et al., 2000). We observed significant deviations of LH firing rates from this impedance-predicted decay (Fig. 4). Understanding the origins of these deviations is an important, but complex, problem for future study. In theory, these deviations can arise from differential expression of many different types of voltage-gated ion channels (calcium, sodium, potassium, or nonselective channels may all contribute: Puil et al., 1986; Hutcheon et al., 1996), as well as from differences in dendritic geometry (Mainen and Sejnowski, 1996). Only a limited knowledge of these parameters currently exists for the different LH cell types (Li et al., 2002; van den Pol et al., 2004; Schöne et al., 2011; Karnani et al., 2013; Romanov et al., 2017). To fully define these parameters in future studies, a comprehensive transcriptomic, pharmacological, and structural comparison of LH cell types and circuits would be necessary, together with modeling approaches.

In terms of physiological significance, the oscillation literature has focused largely on concepts such as input selection and plasticity, consolidation of learned information, representation of phase information, or binding cell assemblies (Buzsáki and Draguhn, 2004). Our results could be considered an example of assembly binding, where, as oscillation frequency is increased, the LH functional assembly shifts from VGAT-GAD65-MCH-orexin to VGAT-GAD65-MCH. Based on known properties of orexin and non-orexin neurons, it is possible to speculate about possible benefits of this shift. One benefit could be to remove an orexin-associated stress state. Orexin activity evokes physiologic hallmarks of stress and creates behavioral aversion (Suzuki et al., 2005; Johnson et al., 2010; Heydendael et al., 2014; Bonnavion et al., 2015). In some contexts, for example eating or formation of food preference driven by VGAT_LH_ and MCH_LH_ neurons, respectively (Domingos et al., 2013; Jennings et al., 2015), it may be important not to associate a stress/aversion signal with food. Consistent with this, *in vivo* recordings from orexin cells show that they are relatively inactive during eating (González et al., 2016). Another, related, benefit would be to create an optimal body state for energy storage. Based on body weight phenotypes resulting from inactivation of the different LH cells, one can view orexin neurons as a natural signal for weight loss, because their inactivation produces weight gain (Hara et al., 2001; González et al., 2016). In contrast, non-orexin neurons can be viewed as a natural signal for weight gain, because MCH and VGAT cell inactivation produces weight loss (Shimada et al., 1998; Whiddon and Palmiter, 2013; Jennings et al., 2015); while chemogenetic LH GAD65 cell activation does not change body weight (our unpublished data). By removing the energy-expending orexin drive, γ oscillations may shift LH output to favor weight gain. An important direction for further research probing causal importance of γ-control of LH cells would be to use some (yet unknown) methods for abrogating the influence of γ oscillations on orexin neurons *in vivo*.

In summary, our study demonstrates an unexpected way of controlling the firing of orexin versus non-orexin LH neurons. Such cell-type-specific LH control was previously thought to be achievable only by cell-type-selective chemical signals, but our results now show that nonselective electrical input can create cell-type-specific effects on hypothalamic firing. This insight opens up new avenues for future research on how this novel control mode can be utilized physiologically via internally-occurring hypothalamic oscillations (Carus-Cadavieco et al., 2017), or, in theory, therapeutically, via a deep-brain-stimulation paradigm promoting a particular oscillation (Maling et al., 2012; Sun et al., 2015). Considering the pivotal role of the LH in physiology and behavior, this reveals an important dimension of controlling the functions and malfunctions of this brain region.

## Synthesis

Reviewing Editor: Douglas Bayliss, University of Virginia School of Medicine Decisions are customarily a result of the Reviewing Editor and the peer reviewers coming together and discussing their recommendations until a consensus is reached. When revisions are invited, a fact-based synthesis statement explaining their decision and outlining what is needed to prepare a revision will be listed below. The following reviewer(s) agreed to reveal their identity: Tamas Horvath. Note: If this manuscript was transferred from JNeurosci and a decision was made to accept the manuscript without peer review, a brief statement to this effect will instead be what is listed below.

Your revised manuscript and responses were re-reviewed, and deemed satisfactory for publication. Congratulations on a very nice contribution.
